# The acute airway inflammation induced by PM_2.5_ exposure and the treatment of essential oils in Balb/c mice

**DOI:** 10.1038/srep44256

**Published:** 2017-03-09

**Authors:** Hetong Wang, Laiyu Song, Wenhui Ju, Xuguang Wang, Lu Dong, Yining Zhang, Ping Ya, Chun Yang, Fasheng Li

**Affiliations:** 1 Dept of Chemistry, Dalian Medical University, Dalian 116044, Liaoning Province, People’s Republic of China; 2 Dept of Immunological and Microbiological Laboratory, Dalian Medical University, Dalian 116044, Liaoning Province, People’s Republic of China; 3 Atmospheric Environment Research Institute, China Research Academy of Environmental Sciences, Beijing 100012, People’s Republic of China; 4 Environmental Monitoring Station of Langfan, Langfang Environmental Protection Bureau, Langfang 065000, Hebei Province, People’s Republic of China; 5 Dept of Nuclear Medicine, The First Affiliated Hospital of Dalian Medical University, Dalian 116011, LiaoNing Province, People’s Republic of China

## Abstract

PM_2.5_ is the main particulate air pollutant whose aerodynamic diameter is less than 2.5 micron. The inflammation of various respiratory diseases are associated with PM_2.5_ inhalation. Pro-inflammatory cytokine IL-1β generated from effected cells usually plays a crucial role in many kinds of lung inflammatory reactions. The exacerbation of Th immune responses are identified in some PM_2.5_ related diseases. To elucidate the underlying mechanism of PM_2.5_-induced acute lung inflammation, we exposed Balb/c mice to PM_2.5_ intratracheally and established a mice model. Acute lung inflammation and increased IL-1β expression was observed after PM_2.5_ instillation. Regulatory factors of IL-1β (TLR4/MyD88 signaling pathway and NLRP3 inflammasome) participated in this lung inflammatory response as well. Treatment with compound essential oils (CEOs) substantially attenuated PM_2.5_-induced acute lung inflammation. The decreased IL-1β and Th immune responses after CEOs treatment were significant. PM_2.5_ may increase the secretion of IL-1β through TLR4/MyD88 and NLRP3 pathway resulting in murine airway inflammation. CEOs could attenuate the lung inflammation by reducing IL-1β and Th immune responses in this model. This study describes a potentially important mechanism of PM_2.5_-induced acute lung inflammation and that may bring about novel therapies for the inflammatory diseases associated with PM_2.5_ inhalation.

Air pollution has become one of the most critical issues in modern society of China^1^,^2^. PM_2.5_ (particulate matter with an aerodynamic diameter no more than 2.5 μm), the main component of air pollution in China, poses a serious hazard to human health. Toxicological studies reveal that PM_2.5_ is likely to attack the airway and circulations^3^,^4^. The toxicity caused by PM_2.5_ is a combined effect of particles and the adsorbed toxic pollutants, such as biological components (endotoxin, pollen, fungal spores, viruses, and bacteria), particles, polycyclic aromatic hydrocarbons (PAHs), volatile organic compounds (VOCs) and heavy metals^5^,^6^,^7^. After exposure, PM_2.5_ would go deeply into the bronchial and invade the fine bronchial and alveolar. The inhalation of PM_2.5_ has been found to be associated with the inflammation of many airway diseases including allergic airway inflammation, asthma and chronic obstructive pulmonary disease (COPD)^8^,^9^,^10^. By investigating the underlying mechanism of PM_2.5_ induced acute lung inflammation, novel approaches may result for preventing and curing PM_2.5_ related inflammatory disease.

Interleukin-1β (IL-1β) is known as a pivotal pro-inflammatory cytokine in orchestrating the local and systemic inflammation of infection and injury^11^,^12^. IL-1β production from effected cells has been found in many kinds of lung inflammatory reactions induced by viruses, bacteria and drugs^13^,^14^. Some *in vitro* studies found that PM_2.5_ could induce the secretion of IL-1β from macrophags through multiple pathways^15^. The activated TLR4/MyD88/NF-κB pathway participates in the expression of pro-IL-1β in the cytoplasm^16^,^17^,^18^. Generation of mature IL-1β requires NLRP3 infammasome and the following caspase-1 activation as well^19^. This pro-inflammatory cytokine also participates in the immune response such as activating macrophages, initiating Th17 response and etc^20^,^21^,^22^.

Essential oils (EOs) are refined from plants and flowers or fruit, leaf, stem, root, through distillation, extrusion or solvent extraction^23^. Evidences display that EOs extracted from Mint, Eucalyptus, Spruce and Frankincense benefit human health in various aspects^24^,^25^,^26^,^27^. Terpenoids, the main component of EOs, exhibits antioxidant, anti-inflammatory and anti-tumor activities in various diseases, including sinusitis, asthma and COPD^28^,^29^. In modern society, people usually use compound EOs (CEOs), mixed by two or more EOs, instead of single EO to achieve better results. Annalucia Serafino *et al*. reported that EOs extracted from Eucalyptus is able to implement the innate cell-mediated immune response^30^. Another *in vitro* study also reveals that EOs play significant role on alveolar macrophages from patients with COPD^31^. Although the effect of EOs on other inflammation has been demonstrated, few studies have investigated the function of EOs in PM_2.5_- induced acute lung inflammation.

In this study, we used a mouse model to evaluate the initiation of an inflammatory response by PM_2.5_. To investigate the underlying mechanism and find a potential cure, we exposed mice to CEOs (compound EOs mixed by isometric Mint, Eucalyptus, Spruce and Frankincense EOs) and assessed the effect of CEOs in the acute airway inflammation induced by PM_2.5_. We observed the change of Th immune response in the acute inflammation and the possible mechanism. The present work demonstrates the important role of IL-1β in acute lung inflammation and suggests a potential underlying mechanism.

## Materials and Methods

### Animals

Male BALB/c mice were purchased from the Changsheng biotechnology Co., LTD. (Shenyang, China) at 6–8 weeks of age. All animals were housed in a specific pathogen-free environment and were maintained on standard mouse chow at an environmental temperature of 24 ± 1 °C and a 12/12 h light/dark cycle with water *ad libitum*. All animal experiments were approved by the Animal Care and Use Committee of Dalian Medical University, which complies with the National Institutes of Health Guide for the Care and Use of Laboratory Animals.

### Reagents

Particulate matters were collected in Langfang (Hebei, China) and supplied by National Environmental Protection Agency. Mint, Eucalyptus, Spruce, Frankincense EOs were supplied by Absolute Aromas Ltd (4 Riverwey Alton GU34 2QL England). All EOs are 100% purity. We mixed isometric Mint, Eucalyptus, Spruce and Frankincense EOs to fabricate CEOs according to aromatherapy. Kits for ELISA were purchased from R&D System (Minneapolis, USA). TLR4 and NLRP3 antibodies for immunohistochemistry were purchased from Sigma Aldrich (USA).

### Analysis of the composition of CEOs

The chemical compositions of CEOs were analyzed by gas chromatography-mass spectrometry (GC-MS). A Hewlett-Pakard (Palo Alto, CA, USA) HP 6890 series plus gas chromatograph coupled to a 5973 mass selective detector was used. The HP-FFAP column for GC was a capillary quartz column with a dimension of 30 cm × 0.25 mm, and film thickness of 0.25 μm (Hewlett-Pakard, Palo Alto, CA, USA). The temperature of the GC system was increased from 60 °C to 150 °C at a rate of 6 °C/min, subsequently raised to 250 °C at a rate of 10 °C/min, and finally maintained at 250 °C for 5 min. As the carrier gas, helium was applied at a flow rate of 1.0 ml/min in a 1.0 μL of injection volume. Mass spectra were recorded under an electron ionization energy of 70 eV within an m/z range of 10–500. The ion source temperature was 230 °C.

### PM_2.5_ collection, analysis and preparation procedure

PM_2.5_ was collected with PM_2.5_ high volume air sampler (Thermon Anderson, USA) using ultra fine quartz fiber filters (General Electric, USA) at a flow rate of 68 m^3^/h for 24 h per day from January to March 2013 in Langfang (Hebei, China). Organic and element carbon was detected by RT-4 carbon analyzer (Sunset Lab, USA). Water soluble ions were measured by ICS-2000/ICS-5000 ion chromatograph (Dionex, USA). The element composition of PM_2.5_ was detected by PE-SciexDR II inductively Coupled Plasma Mass spectromete (PerkinElmer, USA) and PAH was detected by Scion TQ gas chromatograph-mass spectrometer (Bruker Daltonic, USA). The filters were dried (24 h at 50 °C) and weighed respectively before and after PM collection. Filters containing PM_2.5_ were cut and administrated by ultrasonic sonication in sterile distilled water for 2 h. Detached PM_2.5_ was then vacuum-freeze dried, weighed and stored at −20 °C^32^,^33^. Particles were suspended in certain amount of sterile saline to achieve the PM_2.5_ suspension with a concentration of 10 mg/ml for mouse model and PM_2.5_ suspension was always sonicated and votexed before intratracheal instillation.

### Mouse model of PM_2.5_-induced acute lung inflammation and CEOs treatment

All 96 Balb/c mice were randomly divided into four groups (n = 24), as follows: (1) exposure to PM_2.5_ by intratracheal instillation of 50 μl aqueous suspensions of 0.5 mg PM_2.5_ in sterile saline at day 0 and day 2 (PM_2.5_ group); (2) intratracheal instillation of 50 μl sterile saline at day 0 and day 2 (control group); (3) exposure to PM_2.5_ by intratracheal instillation of 50 μl aqueous suspensions of 0.5 mg PM_2.5_ in sterile saline at day 0 and day 2, static inhalation of 200 μl sterile saline with 2 drops of CEOs every day since the day before PM_2.5_ instillation. Mice were administrated by CEOs for 30 min per day until the day before sacrificed (PM_2.5_ + CEOs group)^34^,^35^; (4) exposure to PM_2.5_ by intratracheal instillation of 50 μl aqueous suspensions of 0.5 mg PM_2.5_ in sterile saline at day 0 and day 2, static inhalation of 200 μl sterile saline alone since the day before PM_2.5_ instillation. Mice were administrated by saline for 30 min per day until the day before sacrificed (PM_2.5_ + saline group). Mice exposed to PM_2.5_ underwent direct oral-tracheal instillation of aqueous suspensions of PM_2.5_ as described previously^36^. Mice were sacrificed on days 3, 7 and 14 after intratracheal instillation. Bronchoalveolar lavage fluid (BALF) was collected by cannulating the trachea and then injecting and retrieving 1 ml cold PBS for three times^37^. All samples were centrifuged (1200 g for 10 min.) at 4 °C to obtain the cell pellet. After lysis of red blood cells, the BALF cell pellet was washed twice and re-suspended in PBS and the total cell count was analyzed. Differential cell count was analyzed after Giemsa staining. Macrophages, neutrophils and lymphocytes were identified from fields of 200 cells using standard morphologic criteria.

### Pathological examination

Mice lung tissues were fixed in 4% paraformaldehyde, dehydrated, embedded in paraffin and cut into sections of 4 μm. After deparaffinization, the tissues were stained with hematoxylin and eosin (H&E), and the histopathological lesions and changes were observed under a light microscope. Six random non-coincident microscopic fields were assessed per animal. The analyses were blindly accomplished by two independent researchers.

### RNA extraction and real-time reverse transcription (RT)-polymerase chain reaction (PCR)

Lung and spleen tissues were obtained and total RNA was extracted using TRIzol reagent (Invitrogen, Carlsbad, CA, USA), according to the manufacturer’s protocol. Total lung RNA (1 μg) and spleen RNA (1 μg) samples were managed separately by gDNA eraser at 42 °C for 2 min and then reverse-transcribed (RT) in 20 μl volumes using a program of 37 °C for 15 min and 85 °C for 5 s using T100 Thermal Cycler (Applied BIO-RAD, Hercules City, CA, USA). The reverse transcription process was performed with a RrimeScript RT reagent Kit with gDNA eraser (Perfect Real Time) kit (RR047A; Takara, Dalian, China). Primers were designed using Primer3 (http://bioinfo.ut.ee/primer3–0.4.0/primer3/), and sequences were submitted to BLAST (http://blast.ncbi.nlm.nih.gov/Blast.cgi). Real-time RT-polymerase chain reaction (PCR) was then performed with a SYBR Premix Ex Taq II (Tli RNaseH Plus) kit (RR820A; Takara, Dalian, China). A total of 2 μl of cDNA was used in each 25 μl- PCR volume. Each sample was assayed in duplicate. Differences in amplification efficiencies between the target and housekeeping genes were identified by comparing standard curve slopes. Real-Time PCR was performed with TP800 Thermal Cycler Dice (Applied Real Time System) according to the following program: 95 °C for 30 s, 40 cycles of 95 °C for 5 s, and 60 °C for 30 s. PCR analyses were performed with TP800 system software.

### Immunohistochemical staining

TLR4 and NLRP3 proteins in lung tissues were examined with immunohistochemical (IHC) staining. The samples were fixed with paraformaldehyde at 4 °C for 4 h, washed with PBS containing 20% sucrose for 4 h, embedded and cut into 4-μm-thick sections on acid pre-treated slides. After dewaxing, blocking endogenous peroxidase and repairing the antigen, we incubated the lung tissues sections with rabbit anti-mouse TLR4 and NLRP3 antibodies (1:200 dilution) at 4 °C over night and followed by incubation with an HRP-labeled Goat Anti-Mouse IgG (H+L) as a secondary antibody (1:100 dilution) at 37 °C for 30 min. The results were observed under a light microscope.

### Cytokine analysis by enzyme-linked immunosorbent assay

After BALF and blood obtained, samples were centrifuged at 4 °C. Supernatants and serum were stored at -80 °C for subsequent analysis. Protein concentrations of IL-1β in BALF and IFN-γ, IL-4 in serum were analyzed using ELISA kits according to the manufacturer’s instructions.

### Statistics and software

Statistical Package for the Social Sciences (SPSS) statistical software for Windows (SPSS Inc., Chicago, USA, version 20.0) was used to conduct statistical analyses. Data was reported as means ± standard error of the means (SEM). Data analysis was performed by unpaired t-test, or by Mann-Whitney rank-sum test if data was not normally distributed. *P* < 0.05 was considered statistically significant.

## Results

### The chemical composition of CEOs

Chemical identification of CEOs was accomplished by GC-MS analyses. 25 compounds were identified. The main constituents of CEOs were Eucalyptol (16.7%), α-Pinene (16.54%), Menthol (12.86%) and Cinene (7.37%). The main chemical compositions were shown in Table 1.

### The characterization of PM_2.5_

Compositions of PM2.5 sample was shown in Table 2. The main component was carbon, including organic carbon (58.36 μg/m^3^) and elemental carbon (10.84 μg/m^3^). NH_4_^+^ (29.15 μg/m^3^), NO_3_^−^ (46.04 μg/m^3^) and SO_4_^2−^ (47.90 μg/m^3^) represented the main composition of water soluble ion. Among the 17 metal elements we detected, the content of Ca (4.12 μg/m^3^), Na (1.92 μg/m^3^), Al (1.42 μg/m^3^) and Mg (0.98 μg/m^3^) was very high, meanwhile heavy metal such as Zn (1.74 μg/m^3^), Pb (0.54 μg/m^3^) and Cu (141.08 × 10^–3^ μg/m^3^) was also very high in the ambient PM_2.5_ samples. Among the polycyclic aromatic hydrocarbons (PAH), Fluoranthene (67.38 × 10^−3^ μg/m^3^), pyrene (31.61 × 10^−3^ μg/m^3^) and Benzo [b] fluoranthene (30.26 × 10^−3^ μg/m^3^) represented main components of PAH.

### Acute lung inflammation and IL-1β generation induced by PM_2.5_ exposure

**Figure 1:**
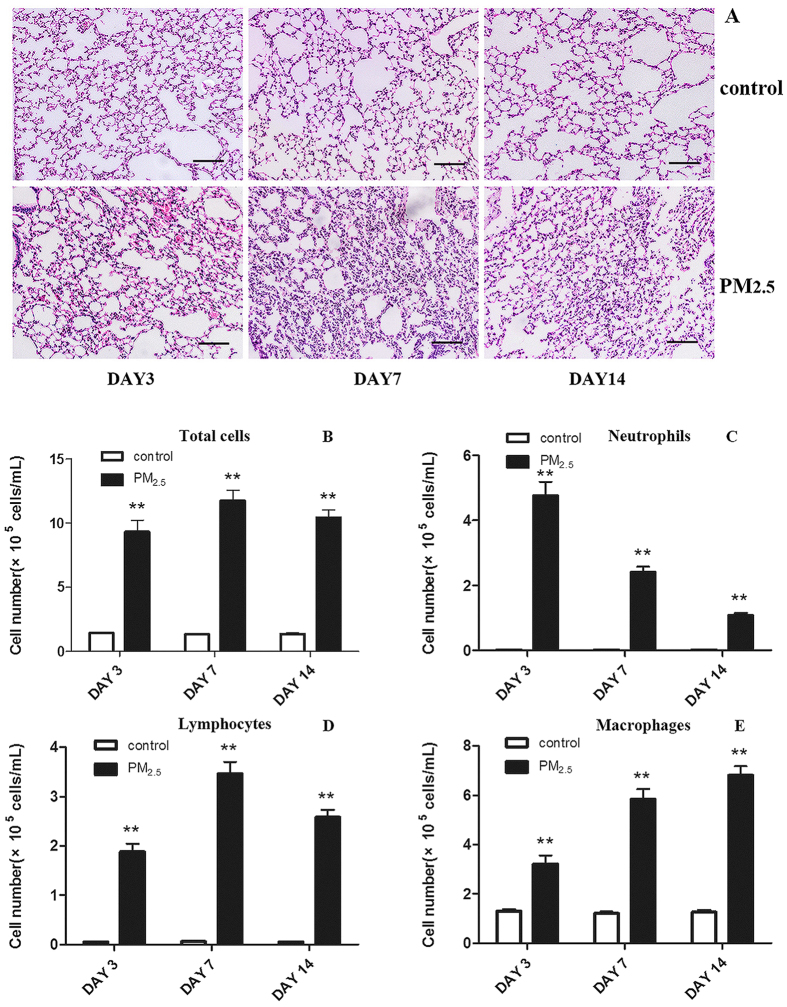
Mice lung tissues pathohistological changes, total cells and differential cells count of BALF after administration by PM_2.5_ or saline (bar = 50 μm). Mice lung tissues were obtained on day 3, 7, 14 after administration by PM_2.5_. Samples were stained using H&E. Histopathological lesions and changes were assessed by histological analyses of six random fields per sample by optical microscope. PM_2.5_ significantly increased cells infiltration and altered alveolar walls structure (**A**). PM_2.5_ facilitated the accumulation of inflammatory cells in BALF. Total cells (**B**), neutrophils (**C**), lymphocytes (**D**) and macrophages (**E**) in BALF were counted using Giemsa staining. Results are represented as means ± SEM (**P < 0.01, *P < 0.05 versus the control group).

To investigate the inflammatory impact of PM_2.5_ on respiratory tract, mice were exposed to PM_2.5_ via intratracheal instillation. Lung tissues and BALF in control group and PM_2.5_ group were collected after mice were sacrificed. Morphological lesions and changes of lung tissue sections were observed using optical microscope. No obvious histopathological alterations were found in control group at all time-points. In contrast, cells infiltration and alveolar walls changes remarkably appeared in PM_2.5_ group at all three time-points, particularly on day 7 (Fig. 1A). Total cells and differential cells count of BALF were also evaluated. Total cells, neutrophils, lymphocytes and macrophages of BALF were significantly increased in PM_2.5_ group compared with those in control group at all three time-points (Fig. 1B–E). Total cells and lymphocytes showed more significant increases on day 7. Meanwhile, in PM_2.5_ group, the neutrophils were decreased and the macrophages were increased with time. The distinct airway inflammatory changes and significantly increased total and differential cells in BALF after PM_2.5_ exposure demonstrated an acute lung inflammation in mice induced by PM_2.5_ exposure.

**Figure 2:**
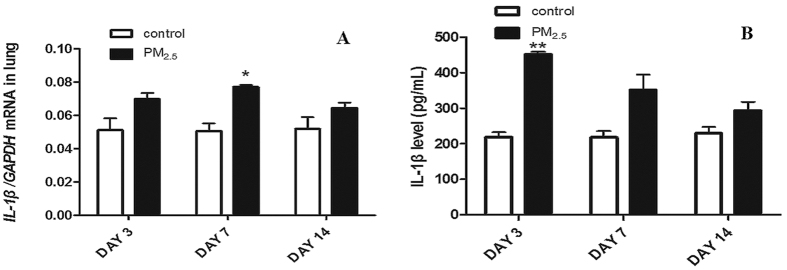
PM_2.5_ upregulated IL-1β mRNA level of lung tissues and IL-1β protein level of BALF. IL-1β mRNA expression of lung tissues (**A**) was assayed by real-time RT-PCR using the ΔCt method. Glyceraldehyde 3-phosphate dehydrogenase (GAPDH) was used as loading controls for mRNA expressions. Levels of IL-1β (**B**) in BALF were detected by ELISA. PM_2.5_ promoted the generation and secretion of IL-1β in lung and BALF respectively. Data are expressed as mean ± standard error of the mean (**P < 0.01, *P < 0.05 versus the control group).

Considering the proinflammatory function of IL-1β in the inflammatory process of various lung diseases, we then examined the mRNA and protein expression of IL-1β in lung tissue and BALF respectively. The mRNA expression of IL-1β in lung tissues was significantly increased in PM_2.5_ group than that in control group, especially on day 7 (Fig. 2A). Similarly, PM_2.5_ significantly increased protein expression of IL-1β in BALF on day 3 and day 7 (Fig. 2B). These results suggested that the large amount of IL-1β generation may involve in the acute lung inflammation induced by PM_2.5_.

### PM_2.5_ may induce the secretion of IL-1β by activating TLR4/MyD88 pathway and NLRP3 inflammasome

The large amount of IL-1β generation in PM_2.5_-induced acute lung inflammation, particular in the early stage of this process, reminds us to investigate the mechanism of its production and secretion. Recent investigations revealed that TLRs activation by PAMPs could lead to pro-IL-1β accumulation in the cytoplasm, which subsequently matured by caspase-1 upon NLRP3 inflammasome activation.

**Figure 3:**
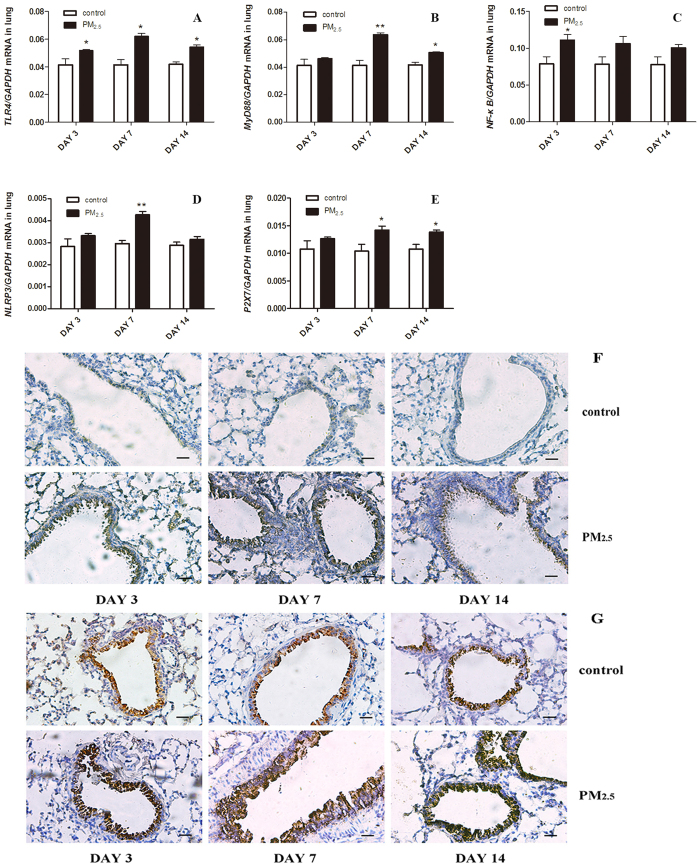
PM_2.5_ increased mRNA expression of TLR4/MyD88 pathway and NLRP3 inflammasome. mRNA levels were assayed by real-time RT-PCR using the ΔCt method. GAPDH was used as loading controls for mRNA expressions. PM_2.5_ increased the expression of TLR4 (**A**), MyD88 (**B**), Nf-κB (**C**), NLRP3 (**D**) and P2 × 7 (**E**) of lung tissues. Data are expressed as mean ± standard error of the mean (**P < 0.01, *P < 0.05 versus the control group). PM_2.5_ increased TLR4 (**F**) and NLRP3 (**G**) protein expression in lung tissues at all time-points, especially at bronchiole section.

Thus we assessed the mRNA expression of key genes in TLR4/MyD88 pathway and NLRP3 inflammasome together with its upstream signal P2X7 of lung tissues. We demonstrated that TLR4, MyD88, Nf-κB, NLRP3, P2X7 mRNA levels of lung tissues were increased in PM_2.5_ group compared with those in control group at all three time-points (Fig. 3). Among these molecules, TLR4 was significantly increased at all time-points meanwhile its adaptor protein MyD88 showed a slight increase on day 3 and obvious increases on day 7 and day14 (Fig. 3A,B). Nf-κB, a down-stream effector of TLR4/MyD88 signaling, was significantly increased on day 3 but slightly increased on day 7 and day 14 (Fig. 3C). P2X7 displayed remarkable increases on day 7 and day 14 (Fig. 3E), moreover, NLRP3 inflammasome just showed a distinct increase on day 7 (Fig. 3D). We also detected TLR4 and NLRP3 protein expression in lung tissues by Immunohistochemistry. As shown in Fig. 3F and G, PM_2.5_ significantly increased TLR4 and NLRP3 protein expression at all three time-points, especially at bronchus. These data provided basis for the hypothesis that TLR4/MyD88 pathway and NLRP3 inflammasome might be activated to generate IL-1β in PM_2.5_-induced acute lung inflammation.

### CEOs may contribute to suppressing PM_2.5_-induced acute lung inflammation

**Figure 4:**
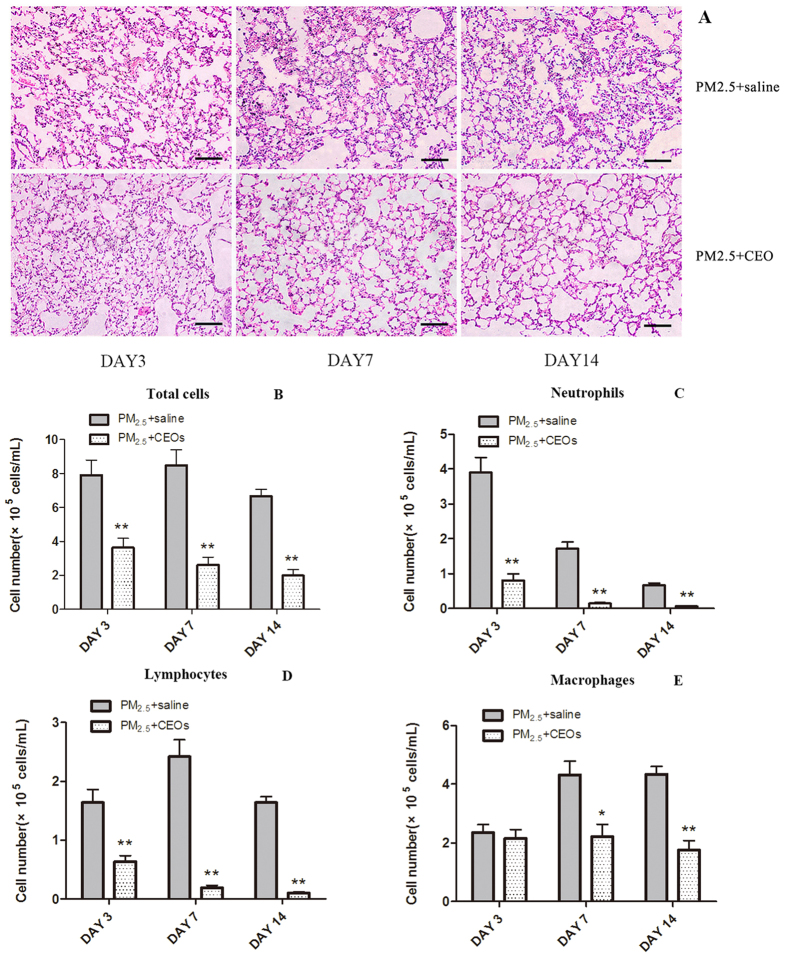
Mice lung tissues pathohistological changes, total cells and differential cells count in BALF after saline or CEO treatment upon PM_2.5_ exposure (bar = 50 μm). Mice lung tissues were obtained on day 3, 7, 14 after administration by PM_2.5_. Samples were stained using H&E. Histopathological lesions and changes were assessed by histological analyses of six random fields per sample by optical microscope. CEOs significantly reduced cells infiltration and alveolar walls alteration time-dependently (**A**). CEOs decreased the accumulation of inflammatory cells of BALF. Total cells (**B**), neutrophils (**C**), lymphocytes (**D**) and macrophages (**E**) of BALF were counted using Giemsa staining. Results are represented as means ± SEM (**P < 0.01, *P < 0.05 versus PM_2.5_ + saline group).

At present, there is no effective treatment for the respiratory and circulatory diseases induced by PM_2.5_. EOs have been deemed to have anti-inflammatory, antifungal and antinociceptive properties in various pulmonary diseases, including pneumonia and lung cancer. To investigate the functions of CEOs in PM_2.5_-induced acute lung inflammation, we respectively administrated with saline alone or CEOs + saline via static inhalation to mice after PM_2.5_ exposure. Lung and spleen tissues, BALF and serum samples in PM_2.5_ + saline group and PM_2.5_ + CEOs group were collected after mice were sacrificed on day 3, day 7 and day 14 upon PM_2.5_ exposure. The histopathological changes of lung tissues were observed by H&E staining. According to the histopathological result, CEOs inhibited PM_2.5_-induced acute lung inflammation time-dependently (Fig. 4A).

**Figure 5:**
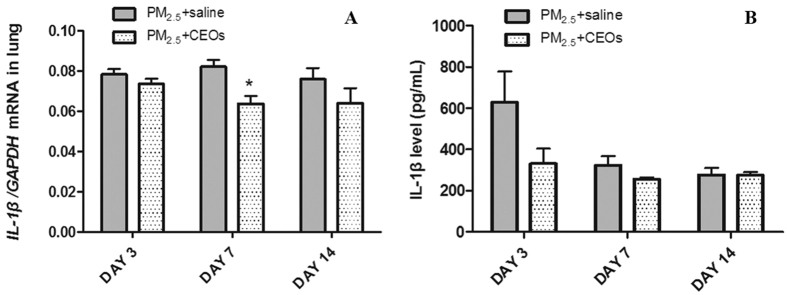
CEOs downregulated IL-1β mRNA level of lung tissues and IL-1β protein level of BALF respectively. IL-1β mRNA expression of lung tissues (**A**) was assayed by real-time RT-PCR using the ΔCt method. GAPDH was used as loading controls for mRNA expressions. IL-1β level (**B**) of BALF was detected by ELISA. Data are expressed as mean ± standard error of the mean (*P < 0.05 versus the PM_2.5_ + saline group).

We subsequently assessed total and differential cells count of BALF in PM_2.5_ + saline group and PM_2.5_ + CEOs group. Total cells, neutrophils, lymphocytes and macrophages of BALF were significantly decreased in PM_2.5_ + CEOs group compared with those in PM_2.5_ + saline group (Fig. 4B–E). Furthermore, we respectively measured IL-1β gene expression of lung tissues and protein expression of BALF. IL-1β mRNA expression of lung tissues (Fig. 5A) and protein expression of BALF (Fig. 5B) were down-regulated in PM_2.5_ + CEOs group than those in PM_2.5_ + saline group at all three time-points, especially at the early stage of inflammatory process. These result indicated that CEOs could attenuate PM_2.5_-induced acute lung inflammation in mice and decrease IL-1β generation in this model.

### CEOs could inhibite the activation of TLR4/MyD88 pathway and NLRP3 inflammasome induced by PM_2.5_

**Figure 6:**
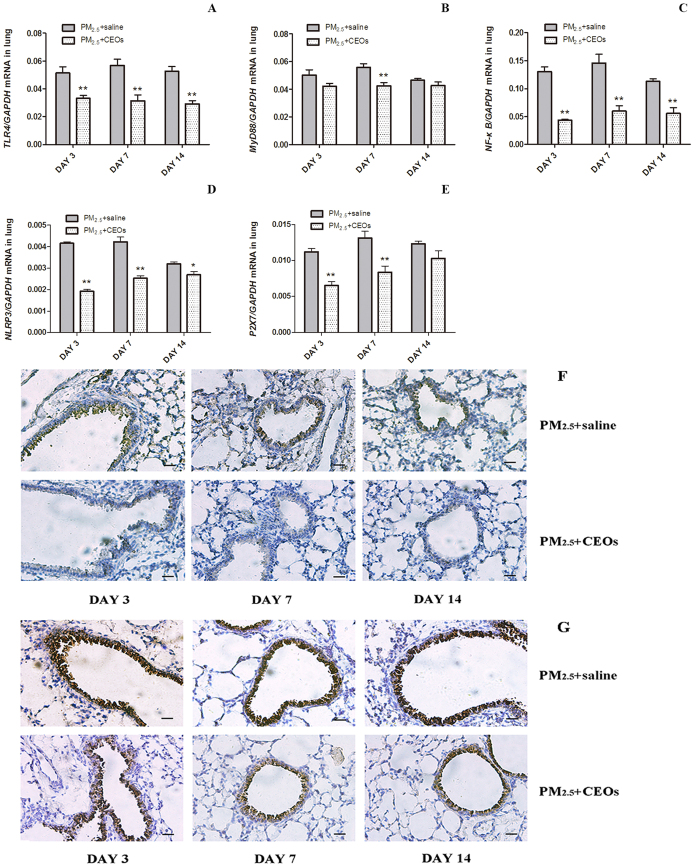
CEOs downregulated mRNA expression of TLR4/MyD88 pathway and NLRP3 inflammasome. mRNA levels were assayed by real-time RT-PCR using the ΔCt method. GAPDH was used as loading controls for mRNA expressions. CEOs downregulated mRNA expression of TLR4 (**A**), MyD88 (**B**), Nf-κB (**C**), NLRP3 (**D**) and P2 × 7 (**E**) of lung tissues. Data are expressed as mean ± standard error of the mean (**P < 0.01, *P < 0.05 versus the PM_2.5_ + saline group). CEOs reduced TLR4 (**F**) and NLRP3 (**G**) protein expression in lung tissues at all time-points, especially at bronchiole section.

To explore the potential mechanism of CEOs in PM_2.5_-induced acute lung inflammation and IL-1β reduction, we evaluated mRNA expression of TLR4/MyD88 pathway and NLRP3 inflammasome on the basis of our previous study. Our data revealed that the TLR4 (Fig. 6A), Nf-κB (Fig. 6C) and NLRP3 (Fig. 6D) expressions of lung tissues were significantly down-regulated by CEOs treatment at all three time-points. Besides, MyD88 expression was significantly decreased on day 7 but inapparently decreased on day 3 and day 14 (Fig. 6B). P2X7 expression showed obvious reductions on day 3 and day 7 but an unnotable reduction on day 14 (Fig. 6E). Then we measure TLR4 and NLRP3 protein expression in lung tissues by immunohistochemistry. CEOs significantly inhibited TLR4 (Fig. 6F) and NLRP3 (Fig. 6G) protein expression time-dependently. Our results elicited that CEOs may play a pivotal role in suppressing PM_2.5_-induced acute lung inflammation by lessening synthesis and secretion of IL-1β via refraining TLR4/MyD88 pathway and NLRP3 inflammasome activation.

### CEOs could modulate Th immune responses in PM_2.5_-induced acute lung inflammation

**Figure 7:**
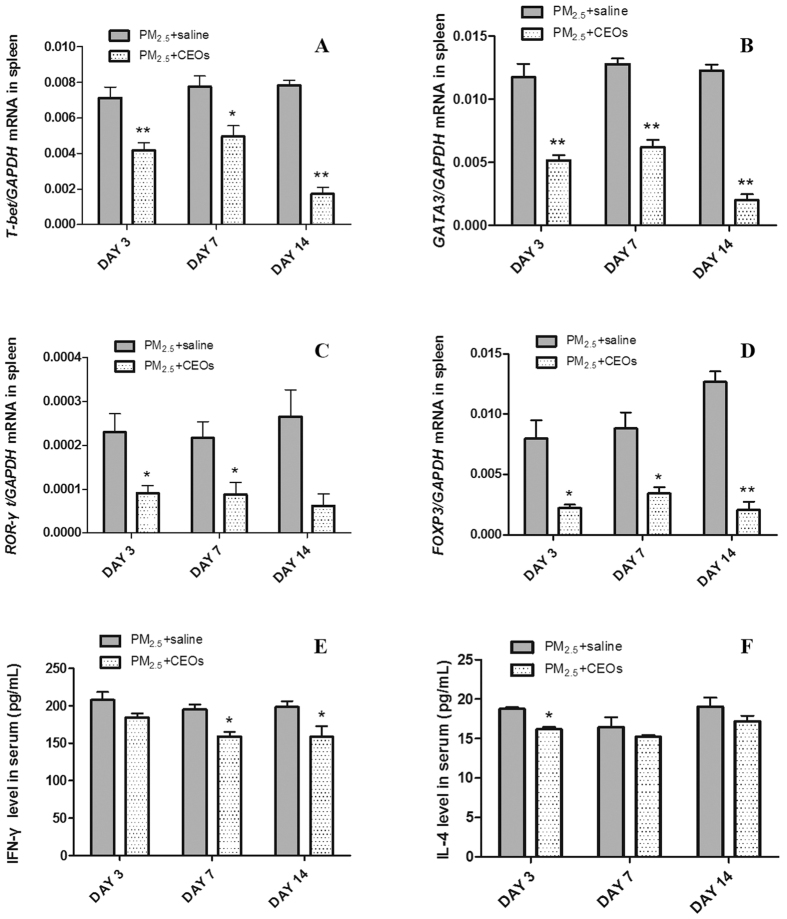
CEOs reduced T-bet, GATA3, ROR-γt and Foxp3 mRNA expression of spleen tissues and IFN-γ and IL-4 protein expressions of serum. mRNA levels were assayed by real-time RT-PCR using the ΔCt method. GAPDH was used as loading controls for mRNA expressions. CEOs significantly reduced expression of the key transforming growth factors of Th1 (**A**), Th2 (**B**), Th17 (**C**) and Treg cells (**D**). Cytokine levels of serum were detected by ELISA. Mice IFN-γ (**E**) and IL-4 (**F**) protein expressions were decreased after CEOs administration. Data are expressed as mean ± standard error of the mean (**P < 0.01, *P < 0.05 versus the PM_2.5_ + saline group).

Many pulmonary diseases are associated with or directly result from aberrant immunological responses. To investigate the functions of CEOs in reducing PM_2.5_-induced acute lung inflammation, we obtained mice spleen tissues to assess mRNA expression of T-bet, GATA3, ROR-γt and Foxp3, the key transforming growth factors respectively belong to Th1, Th2, Th17 and Treg cells. According to the results, T-bet, GATA3, ROR-γt and Foxp3 mRNA expression in PM_2.5_ + CEOs group were significantly down-regulated at all three time-points compared with those in PM_2.5_ + saline group, particularly on day 14 (Fig. 7A–D).

We obtained mice serum and measured levels of IFN-γ and IL-4, the signature cytokines characterizing Th1 and Th2 immune responses. Our data showed that IFN-γ protein expression of serum in PM_2.5_ + CEOs group was significantly down-regulated on day 7 and day 14 (Fig. 7E) meanwhile IL-4 protein expression of serum in PM_2.5_ + CEOs group was significantly reduced on day 3 (Fig. 7F). The results implied that CEOs might decrease the inflammatory response by modulating Th immune responses in mice exposed to PM_2.5_.

## Discussion

PM_2.5_, as the major air pollutant in China, has been demonstrated to increase the incidence of abundant pulmonary and cardiovascular diseases in last decade according to its aerodynamic character^38^,^39^. In this study, we utilized the PM_2.5_ collected from Hebei province (one of the most polluted areas in China) and examined the composition, especially the toxic heavy metals and aromatic hydrocarbons. These components may partially explain the hazard of PM_2.5_. Although PM_2.5_ has been receiving an extensive attention due to its effect on human health, little *in vivo* study elucidates the underlying mechanisms of acute lung inflammation induced by PM_2.5_ inhalation. In this study, we used Balb/c mice to evaluate the potential mechanisms of acute lung inflammation induced by PM_2.5_. The pro-inflammatory cytokine IL-1β and the related regulatory factors of IL-1β in this model were also investigated. CEOs administration decreased PM_2.5_-induced acute lung inflammation significantly. The anti-inflammatory effect of CEOs might be achieved by reducing the inflammatory cytokine and Th immune responses in this model.

We first examined the initiation of PM_2.5_-induced acute lung inflammation. After twice PM_2.5_ treatment, obvious inflammatory changes of lung tissue and BALF could be observed very quickly. Rossella Bengalli *et al*. have proven that PM_10_ infection could trigger release of IL-1β in mice^40^. Then we investigated the mRNA and protein expression of IL-1β in the inflammatory position. Consistent with our speculation, PM_2.5_ significantly increased IL-1β production in murine lung tissues and BALF at three time-points. The rapid rise of the proinflammatory cytokine IL-1β in the early time point suggests a crucial role of IL-1β in this PM_2.5_-induced acute lung inflammation. We examined the signaling pathway responsible for the generation and secretion of IL-1β in this model in the next step. TLRs in host cells could sense the stimulus and induce the synthesis of pro-IL-1β^41^. Some *in vitro* studies also revealed that TLR4 mainly contributed to cytokines production induced by PM_2.5_^42^. Activated NLRP3 inflammasome activates caspase-1 which subsequently cleaves pro-IL-1β into the mature form, IL-1β^43^,^44^. The pro-inflammatory cytokine IL-1β then attracts macrophages, neutrophils and mast cells leading to inflammatory response. Our results of TLR4 and related genes revealed that production of IL-1β is highly possible on account of the activation of TLR4/MyD88 signaling pathway and NLRP3 inflammasome in PM_2.5_-induced acute lung inflammation. However, the precise pathogenic mechanisms of PM_2.5_ still remain poorly understood and knock-out strategies and neutralizing antibodies may contribute to the further investigations.

Studies have confirmed the anti-inflammatory, antifungal and antioxidant effects of EOs on various diseases^45^,^46^,^47^. In present reports, CEOs which mixed with two or more essential oils in certain proportion display better effects than simplex EOs due to synergic interactions. Considering the fact that there is no clear and effective treatment to the acute lung inflammation induced by PM_2.5_ until now, we postulated CEOs might play a role and become a novel therapy in this model. In our study, mice were respectively administrated the CEOs or saline via static inhalation after PM_2.5_ exposure. CEOs significantly restrained PM_2.5_-induced acute lung inflammation time-dependently. We then investigated the feasible mechanism. The decreased mRNA and protein level of IL-1β implied that CEOs may inhibit inflammatory reactions through IL-1β reduction. The administration of CEOs also suppressed IL-1β related signaling pathway. These results also remind us about the importance of TLR4/MyD88 signaling pathway and NLRP3 inflammasome in IL-1β production and PM_2.5_-induced acute lung inflammation.

Previous study suggested the crucial role of immune system in host defense against pathogens and infections^48^. However, excessive and massive immune responses will usually lead to local or systemic inflammation^49^. The exacerbation of Th immune responses were identified in varies PM_2.5_ related inflammatory diseases^50^. Considering the immunosuppressive function of EOs, we speculated that the anti-inflammatory curable role of CEOs may be achieved by manipulating the Th immune responses as well. To better understand the anti-inflammatory mechanism of CEOs, we measured the key transcription factors of Th subsets respectively. CEOs remarkably down-regulated the transcription factors mRNA of Th1 and Th2 cells in the spleen of mice. In addition, CEOs administration decreased Th1 and Th2-related cytokines (IFN-γ and IL-4) in mice serum as well. These data verified the function of CEOs in modulating Th1 and Th2 immune responses in this model. We further explored CEOs effect on Th17 and Treg cells. Key transcription factor of Th17 cells, ROR-γt level was decreased by CEOs inhalation. Interestingly, Foxp3, key transcription factor of Treg cells, which is essential for maintaining self tolerance and limiting inflammatory diseases was reduced as well. These data suggests a novel therapeutic method to PM_2.5_-induced inflammatory diseases and reminds us to further investigate the complex anti-inflammatory mechanisms of CEOs in this model.

In summary, we demonstrated that acute lung inflammation induced by PM_2.5_ may be due in part to the generation of IL-1β. Activation of TLR4/MyD88 signaling pathway and NLRP3 inflammasome may involve in this process. CEOs mixed with Mint, Eucalyptus, Spruce and Frankincense essential oils can inhibit PM_2.5_-induced acute lung inflammation and down-regulating IL-1β production via suppressing activation of TLR4/MyD88 signaling pathway and NLRP3 inflammasome. CEOs can attenuate PM_2.5_-induced acute lung inflammation through regulating Th immune responses as well. The present study describes a potentially important mechanism of PM_2.5_-induced acute lung inflammation that may lead to novel therapies for PM_2.5_-related inflammatory diseases.

## Conclusions

Our present study found that short-term exposure of PM_2.5_ induces acute lung inflammation in Balb/c mice. The increase of IL-1β production in PM_2.5_-induced acute lung inflammation might be associated with the activation of TLR4/MyD88 signaling pathway and NLRP3 inflammasome. In addition, CEOs mixed with Mint, Eucalyptus, Spruce and Frankincense EOs reduce PM_2.5_-induced acute lung inflammation and inhibit murine systemic immune responses. These findings might provide potential mechanisms and a novel therapy of PM_2.5_-related respiratory diseases.

## Additional Information

**How to cite this article:** Wang, H. *et al*. The acute airway inflammation induced by PM_2.5_ exposure and the treatment of essential oils in Balb/c mice. *Sci. Rep.* **7**, 44256; doi: 10.1038/srep44256 (2017).

**Publisher's note:** Springer Nature remains neutral with regard to jurisdictional claims in published maps and institutional affiliations.

## Figures and Tables

**Table 1:** The content of main chemical compositions in CEOs.

Component	Content	Component	Content
Eucalyptol	16.70%	*p*-Mentha-1,4-diene	1.07%
*a*-Pinene	16.54%	*p*-Cymen-8-ol	0.88%
Menthol	12.86%	Globulol	0.67%
Cinene	7.37%	Piperitone	0.63%
*β*-Pinene	5.88%	Isomenthone	0.57%
Menthone	5.74%	Terpineol acetate	0.57%
Bornyl acetate	2.66%	Verbenone	0.49%
Camphene	2.15%	Myrtenol	0.47%
*γ*-Terpinene	1.67%	*trans*-Caryophyl1ene	0.39%
*α*-Terpieol	1.61%	Calamenene	0.39%
*p*-Cymene	1.33%	2-Decanol	0.32%
Caryophyllene	1.21%	*trans*-Carveol	0.32%

**Table 2:** Mean concentration (μg/m^3^) of main compositions detected in PM_2.5_ sample.

	Component	Concentration	Component	Concentration
Carbon	organic carbon	58.36	elemental carbon	10.84
Water soluble ion	Na^+^	1.72	NH_4_^+^	29.15
	K^+^	6.51	NO_3_^−^	46.04
	Ca^2+^	3.78	SO_4_^2−^	47.90
	Mg^2+^	0.68	Cl^−^	10.60
Metal element	Na	1.92	Ni	11.36 × 10^−3^
	Mg	0.98	Cu	141.08 × 10^−3^
	Al	1.42	Zn	1.74
	Ca	4.12	As	65.18 × 10^−3^
	V	7.56 × 10^−3^	Se	14.64 × 10^−3^
	Cr	6.10 × 10^−3^	Mo	6.22 × 10^−3^
	Mn	0.46 × 10^−3^	Pb	0.54
	Fe	1.52 × 10^−3^	Cd	7.12 × 10^−3^
	Co	4.48 × 10^−3^		
Polycyclic Aromatic Hydrocarbons (PAH)	naphthalene	2.15 × 10^−3^	chrysene	22.65 × 10^−3^
	acenaphthylene	0.53 × 10^−3^	Benzo [a] anthracene	15.13 × 10^−3^
	acenaphthene	0.70 × 10^−3^	Benzo [b] fluoranthene	30.26 × 10^−3^
	fluorene	0.98 × 10^−3^	Benzo [k] fluoranthene	3.25 × 10^−3^
	phenanthrene	18.26 × 10^−3^	Benzo [a] pyrene	6.45 × 10^−3^
	anthracene	1.60 × 10-3	Diphenyl [a,h] anthracene	3.82 × 10^−3^
	fluoranthene	67.38 × 10^−3^	Indene [g, h, I] pyrene	10.57 × 10^−3^
	pyrene	31.61 × 10^−3^	Indene [1, 2, 3 -c, d] pyrene	10.92 × 10^−3^
